# A Lay Ethics Quest for Technological Futures: About Tradition, Narrative and Decision-Making

**DOI:** 10.1007/s11569-016-0273-2

**Published:** 2016-08-17

**Authors:** Simone van der Burg

**Affiliations:** IQ Healthcare, Radboud University Medical Center, PO Box 9101, Route 114, 6500 HB Nijmegen, Netherlands

**Keywords:** Narrative, Ethics, Quest, Choice, Nanotechnology

## Abstract

Making better choices about future technologies that are being researched or developed is an important motivator behind lay ethics interventions. However, in practice, they do not always succeed to serve that goal. Especially authors who have noted that lay ethicists sometimes take recourse to well-known themes which stem from old, even ‘archetypical’ stories, have been criticized for making too little room for agency and decision-making in their approach. This paper aims to contribute to a reflection on how lay ethics can acquire more practical relevance. It will use resources in narrative ethics to suggest that in order to be relevant for action, facilitators of lay ethics interventions need to invite participants to engage in a narrative quest. As part of a quest, lay ethicists should be asked to (1) reflect on a specific question or choice, (2) use diverse (imaginative) input which is informative about the heterogeneity of viewpoints that are defended in society and (3) argue for their standpoints.

Scholarly attention to the societal value of new and emerging technologies, responds to an awareness that science and engineering may not only lead to technological products which are unambiguously *good.* While research and development of these technologies goes together with promises about how they will enhance economic growth, strengthen international competitiveness and enlarge human wellbeing, there is awareness of the ways in which they may also, at the same time, put current moral convictions and routines under pressure. Given this uncertain and debatable value, it is considered wise to anticipate the future and determine ahead of time whether technologies that scientists research and develop are actually desirable or not.

One of the ways to do this, is by involving members of the public in an evaluative reflection about new technologies. Such endeavors are sometimes called ‘lay ethics’. Participants in such activities are ‘lay’ in the sense that they received no previous ethical training, yet they are invited to make their values explicit, imagine how those values may be effected by the introduction of a new technology into their lives and evaluate the desirability of those technologies.

These last years, the reflections of lay ethicists are sometimes preferred to the reflections of professional ethicists for two reasons. First of all, the reflections of laymen are rich and take into account the profound and varied ways in which technologies—such as internet or the mobile phone—may alter people’s everyday experience, emotion, motivation to act, daily routine, and ways to (inter)act and relate to others [5, 6, 25]. By contrast, professional ethicists, especially those who adopt a principalist approach grounded in deontology or utilitarian theory, focus just on a selection of general issues, such as risk, autonomy, privacy and justice. These professional ethicists, furthermore, often translate the principles which shape their approach into a general checklist, which significantly shrinks the amount of topics that they are able to take into account and ignore all the other ways in which technologies may have different effects on diverse socio-cultural contexts [13, 1]. It is because the ethical reflection of lay ethicists did not yet pass the filter of ethical theory, that it is considered more apt to take broader, richer and more substantive issues into account.

Another, second, reason to ask laymen rather than professional ethicists is political. It is considered problematic that professional ethicists acquire a privileged position in the debate about the value of new technologies; for, as professionals, their viewpoints carry the weight of expert discourse and they are granted visibility and legitimacy which the viewpoints that non-professional ethicist lack. Therefore, not every participant in the moral world has equal power to give shape to the ethical debate about the desirability of a new technology, while they do all have to live with the effects of that technology. To make the debate more inclusive, scholars have selected members of the general public as the eligible ethical thinkers, therewith making voices audible that would otherwise less likely be heard. Including lay ethicists is expected to ‘increase public trust’ in science, as no one acquires a privileged role in steering developments in a particular direction ([6], 3).

Lay ethics is supposed to be relevant for choice, for it should help to steer the development of new technologies in more desirable directions. Yet, in practice, it does not always succeed to serve that goal. It has been noted, more often, that public discussion about new technologies often repeats and reaffirms well-known moral points of view in a somewhat mantra-like fashion [21, 28]. This happens also in lay ethics interventions. Lay ethics studies by Sarah Davies and Phil Macnaghten are criticized for this reason. While one of the key points in the study by Davies and Macnaghten is to make room for the agency of a lay public in the academic and political debate which usually ignores the public opinions, the themes that participants brought forwards in focus groups are analyzed in terms of old stories, which they call ‘archetypical stories’. This allows to appreciate the deep rootedness of these stories in the cultural history of society. However, the repetitiveness of the same age-old stories also raises the question whether lay ethics is actually able to offer assessments that provide a helpful guide to take action and shape the future.^1^

This paper aims to contribute to the discussion about whether and how the past can be made relevant for present-day decision-making which shapes the future in lay ethics. It will describe the main message of the papers in which Davies and Macnaghten present their lay ethics interventions, as they are the ones who gave shape to a lay ethics that roots in historical stories, and it will describe some of the criticism Davies and Macnaghten received. After that, it will use resources in narrative ethics to show how the relation between tradition, narrative and decision-making towards the future has been understood and discussed there for some decades. It will resume some aspects in that discussion and draw attention to the notion of a ‘narrative quest’, which plays a pivotal role in linking traditional stories to present decision-making and a yet to be experienced future in narrative ethics. Based on this input, the paper will make suggestions as to how that narrative quest can be integrated into lay ethics in order to make it more practically relevant for navigating the future, even if participants bring forwards historically rooted stories.

While I focus here on Davies and Macnaghten’s lay ethics interventions, the aim of this article is broader than just adding to the debate about their studies. It will contribute to a further elaboration of the theme of narrative in assessments of the future, which has recently been called for.^2^ The reason why Davies and Macnaghten are taken here as a starting point is because their approach allows to show how convincing it is to think of narratives as transcending present-day people, while at the same time revealing the difficulties that it raises for an understanding of the agency of lay ethicists.

## Debate about the ‘archetypical stories’

The lay ethics interventions that Sarah Davies and Phil Macnaghten carried out are based on two projects: one was the DEEPEN project, a multidisciplinary project, which involved members of the public from Portugal and the UK into an ethical reflection on nanotechnological futures [5]; the other reports about a similar study carried out in the UK only [18]. In both studies, the participants’ reflection was generated by asking them to reflect on general *optimistic* as well as *pessimistic* narratives about nanotechnology futures, presenting nanotechnology as a science that can contribute to scientific breakthroughs, to human enhancement (more health, lengthening of life and a strengthening and broadening of human capacities), to a cleaner environment, and to different products, but also to control over nature, uncertainty and risks ([5] 142, [18] 29). The DEEPEN project used a 3-stage methodology. First, there was an initial focus group where participants discussed the different representations of nanotechnology which were introduced to them. Second, the focus group would reconvene a few days later and they would discuss the key issues and work on the presentation of these issues in the form of a sketch. Later that day, third, each group would present its sketch in a theatre session. The theatre session was shaped in the tradition of the Brazilian dramatist Augusto Boal ([5] 144). In the UK project, the reflection of the participants was enhanced by confronting them with three general styles of thought that are used to present nanotechnological futures in policy reports, newspaper articles, documentaries, industry presentations and campaign materials ([18] 26).

In both studies there were diverse themes continually recurring in the diverse responses of the focus group participants which, according to the authors, referred to common narratives that seemed to ‘underpin’ them. As these narratives recall stories that have been told and retold in many societies in Europe and the Americas, Davies and Macnaghten call them ‘archetypal stories’ or ‘master narratives’, using terminology by Agnes Heller. Archetypical narratives are ‛deeply rooted within contemporary culture’; they are sometimes referred to as ‘mythic cultural tropes’ because the recurrence of the themes in the speeches of various lay ethicists suggest that they have traditional or legendary status ([5] 146), and sometimes they are explained as offering ‘references to a shared tradition’, which reveals that they not only give expression to a shared way of thinking about the future, as ‘guides of imagination’, but also to a shared way of life ([5] 145). Five examples of such archetypal or master narratives are described in the article about the DEEPEN project; the UK project carried out by Macnaghten alone mentions just a subset of them ([18] 31):

- The ‘be careful what you wish for’ narrative alludes to ancient stories that call for prudence with regard to the immediate force of desires, which may on second thought not be as desirable as was initially thought. Nanotechnologies represent seductive futures, but indulgence in these temptations may have harmful consequences
- The ‘messing with nature’ narrative which summarizes concerns around nanotechnology’s potential to change nature. Nature in this narrative is taken to imply an order, which should not be ‘messed with’ because took the entire evolution to come into being.
- The ‘Pandora’s box’ narrative, which alludes to the story of a temptingly closed box which will release evils if it is opened. It refers to notions of uncertainty with regard to new technology, while it may bear a very attractive promise, it may eventually lead to disaster, and then there’s no turning back.
- The ‘kept in the dark’ narrative, which reveals a feeling of deep powerlessness of the general public in the face of the development of nanotechnology. Not only were many people unaware of the existence of nanotechnology; they also have little way of having any impact on it at all.
- The ‘rich get richer and the poor get poorer narrative’, which draws together concerns about technological development which acquires shape in a context in which commercial and consumption oriented considerations are dominant. The story gives voice to the supposition that technology will benefit those who can pay for it, and not the poorer people.

While these narratives are presented as the result of focus group discussions, naming them ‘archetypical’ or ‘master’ narratives also gives them a meaning that encompasses an analysis of what these people said. They refer to a common history, a lived tradition, which suggests that they will probably remain important in the future. This is also what the authors seem to think. They claim that the catalogue of ‘archetypical narratives’ is open to expansion, for some ‘archetypical stories’ (such as Pandora’s box) are very old, while others (such as ‘the rich get richer and the poor get poorer’ narrative) developed more recently; and they also mention that all of these narratives are counter narratives which oppose the language which has been most dominant since the enlightenment and which holds science responsible for ‘progress’ and ‘breakthroughs’. Yet, the familiarity of the articulated ‘master narratives’, their repetition in ancient stories, in fables, literature, and more recently in films, science fiction and video games is also a reason for the authors to claim that they will be important in the future too: ‘(..) it is precisely these kinds of narrative that will continue to shape popular perceptions of science and technology, and which will provide the landscape over which future technopolitics will be articulated’ ([18] 33).

The historical rootedness of these master narratives sketches a picture of lay ethical reflection as being firmly tied to a shared heritage of stories. While the stories offer resources to adopt a general critical attitude towards new technologies, they also reveal the public’s impoverished agency in shaping technological futures as these stories are inherited and retold rather than shaped by the public. This limited agency is also what came forwards in criticisms of these ‘archetypical stories’ as output of a lay ethics intervention. The optional character of the conversations between the lay ethicists, for example, is reason for Ferrari and Nordmann [9] to call such interventions ‘morality play’, as they rehearse, reiterate and reaffirm the moral points of views ‘in a somewhat ritualistic fashion’ ([9] 173). While such ‘morality play’ is not worthless, Ferrari and Nordmann also criticize it for remaining ‘conversational’, meaning that ‘…concerns are expressed in open-ended ways (..) no decisions need to be taken, no judgments need to be made, no conclusions need to be reached’ ([9] 174). The conversation lacks practical relevance and therefore Ferrari and Nordmann suggest that ‘… nanoethics needs to delineate and sharpen issues in such a way that they can return to the political arena’ ([9] 175).

This criticism receives a different articulation in a recent article by Erik Thorstensen [31]. While part of Thorstensen’s critique of Davies and Macnaghten’s approach to lay ethics is methodological, his somewhat aggressively formulated attack becomes most interesting when he addresses the topic of narrative. Calling the narratives ‘archetypical’ rather than just using them as ‘interpretative tools’ to make sense of the responses of the focus group participants, deserves to be looked at with caution, Thorstensen claims. As ‘archetypical narratives’, these stories become the ‘beginnings and endings of a culture’ and they eclipse the role of story-tellers ([31] 229). This is problematic, according to Thorstensen, because it disrespects the very common presupposition in narrative studies that there are no stories without narrators; moreover, it seems to commit the ‘ontological fallacy’ that supposes that there are stories ‘out there’, quite independent of story tellers and prior to the narrative process ([31] 230). It is of course questionable how a story without a teller can ever offer a valuable lead for action that helps to shape the technological future of society, as the agents who might take such actions are omitted.

Macnaghten and Davies respond to these criticisms with colleague Matthew Kearnes and elaborate their perspective to narrative in later articles. [12, 19]. In an article from 2015, they argue against critics who state that archetypical narratives have little impact on policy, that this is not a limitation of these narratives themselves; rather, it reveals a resistance to these stories by its recipients, such as policy makers who decide about what research should be funded and which products should be allowed on the market. Rather than relying ‘on the dominant master narrative of progress and scientific breakthrough (..)’ ([19] 13), the authors argue here that more responsible research and innovation policy makers should cultivate their capacity to respond to the wider range of considerations that figure in the counter-narratives that lay ethicists bring forwards, which would require ‘institutional and regulatory redesign’ ([19] 14).

In this article, furthermore, Macnaghten, Davies and Kearnes respond to the criticism by an elaboration of their perspective on narrative. They take distance from individual—psychological and cognitive—approaches to narrative, which understand narratives to originate from individual cognitive or affective processes; and they also argue against conversational approaches which take narratives to arise from conversational interaction between people. Instead, they take a perspective which ‘transcends’ individuals as well as immediate interaction between people, for they want to pay attention to how public concerns emerge from social and cultural processes. It is for this reason that they adopt a perspective which allows to appreciate how narratives are based on a discursive, social, cultural and theological ‘heritage’ which people share and which steers the perceptions and opinions that they have individually or which they may bring forwards in conversation with others ([19] 3). Siding with philosopher Charles Taylor, they defend the view that stories depict a world of moral wisdom that is rooted in an inherited and shared past, which encompasses the consciousness of present-day participants in an ethical reflection. Yet, they also leave room for imaginative, and subtle, change. Changes may come about when people articulate the age-old stories in the course of a conversation. As the authors write ‘…narratives, as we understand them, are never static but are always shaped by the moments of enunciation’([19] 7).

Macnaghten, Davies and Kearnes certainly have a point when they argue that agency needs to be granted to tellers of a story by its recipients in a social context—in this case policy makers—and they do make space for tellership and agency in their perspective to narrative. It remains, however, unclear how large that space is. While they state that stories ‘are shaped’ in the process of telling them, they do not make clear how, or on the basis of what, tellers can possibly do that. Macnaghten and his colleagues continue to talk about tellers in somewhat passive phrases, therewith giving the impression that tellers are shaped by the stories, rather than the other way around. It is, therefore, unsurprising that they suspect that ‘enduring narratives exist’, which ‘may provide publics, consciously or not, with the cultural resources to develop an imagination of the social issues associated with emerging technology...’ ([19] 8).^3^

It is unsatisfying, however, to leave it at that. I think Davies, Macnaghten and Kearnes are very right to claim that stories are not invented on the spot by the people who take part in lay ethics interventions. There are, however, resources available in narrative ethics which allow to elaborate this perspective to narrative a few steps further. Their approach to narrative is very similar to perspectives that have been put forwards by communitarian virtue ethicists during the past decades and which have attracted a lot of debate. Charles Taylor is one of these ethicists who is sometimes called ‘communitarian’ because he, just like some others, takes the historical past of a community to be informative for present day moral standpoints. As narrative plays a prominent role in the accounts that these ethicists give of how a person or a social community can be characterized as ‘the same’ at several moments in time, they are also sometimes referred to as ‘narrative ethicists’. In the following, some elements of the debate will be resumed that the perspective by Taylor and others attracted, in order enrich the approach by Macnaghten, Davies and Kearnes and point towards ways to at the same time accept that stories are rooted in the past and transcend individuals and groups of present-day debaters, while also leaving room for tellers to *actively shape the story they inherited* and talk intelligently about choices for the future.

## Narrative ethics and the debate about tradition

The ‘transcendent’ perspective to stories was brought forwards most prominently in the 1980’s by two virtue ethicists Alasdair MacIntyre and Charles Taylor, who argued against the individualism in deontological and utilitarian forms of ethics, which were developed during the Enlightenment [16–17, 30]. The well-known story about the Enlightenment^3^ is that people were liberated from the oppressive powers of church and sovereign, which was supported by the theoretical development of an ethics which laid the power of moral decision-making in the hands of individuals; that is, in their capacity to *reason* (Kant; deontology) or in their capacity to *experience* the effects of actions on people’s wellbeing (Bentham; utlititarianism). MacIntyre and Taylor became famous for saying that this liberating project has a downside; the moral vocabulary of people impoverished (like, in a checklist approach), because the individuals charged with the task to find appropriate moral measures in their own reason or experience, lost the connection to each other as well as to the moral language that provided them with historical resources to think about their lives and the choices they have to make. The work by MacIntyre and Taylor reveals their attempts to restore the connection to that shared past. While MacIntyre takes a radical stance and discards any approach to ethics that isolates individuals from the social context that provides them with meaningful moral distinctions, Charles Taylor provides a more heterogeneous account which includes deontology and utilitarianism in the rich traditional background of contemporary inhabitants of democratic societies in the West.

Both MacIntyre and Taylor, however, presuppose that the social values inherited from the past transcend the individual as well as the immediate interaction between people in the here and now, just like Macnaghten and his colleagues do. MacIntyre metaphorically explains that for newcomers in a particular social community that already uses moral distinctions, it is like being put on a stage and becoming part of a play that they did not invent themselves; and Charles Taylor uses a spatial metaphor saying that seeking moral orientation is like the search for spatial orientation: both take place in a space that was already there before they arrived. Just like people could not come to reflect that ‘… since they were spatial beings, they ought after all to develop a sense of up and down, right and left, and find landmarks which would enable them to get around’, they likewise cannot imagine living without moral discriminations as they were always already living in a ‘moral space’ ([30] 31). It is therefore of little use to try to find the source of value within oneself, for it ‘belongs to the class of the inescapable’ for human beings ‘(..) to exist in a space of questions about strongly valued goods, prior to all choice or adventitious cultural change’ ([30] 31). Without a space of questions about what ‘good’ and ‘bad’ is, which took shape in a history of interaction, an individual would not know where to begin thinking about what it would be sensible to do or to be. Any individual who would try to do this, according to Taylor, ‘would be outside our space of interlocution; he wouldn’t have a stand in the space where the rest of us are. We would see this as pathological’ ([30] 31).

Quotes such as these have led to a lively debate about whether and how much individuals can change the moral discriminations that define the moral spaces in which they seek their initial orientation in life. This is a question that concerns the individual, as well as society; for, individuals are the present participants in that society and as such, they are the ones to decide what moral distinctions that they inherited from the past deserve to be preserved and which ones ought to be changed for the coming generations. Both MacIntyre and Taylor explicitly state that it is possible for individuals to revise the morality that they inherited from past generations. They preserve the agency of the tellers of stories, by ascribing to them the power to co-create the storyline that relates past to future and which may narrate a history of change. Considerations about what aspects of past morality deserve continuation and what aspects should be revised are part of what they call a ‘narrative quest’ ([16] 219, [30] 48). A narrative ‘quest’ differs from ‘research’ in the sense that it does not focus on cognitive knowledge which aims to be verifiable in all times and places; a narrative quest roots in concrete life experiences of particular people.

A quest may be understood individually, but also socially. Socially, it connects the past history of a concrete human interaction or ‘practice’—say, rearing children, farming, architecture or medicine—with the future by telling a story about past experiences and how perspectives generated from them on the goals that should be realized in the future or in what ways those goals should be realized. Individually, it is the result of a person’s attempt to navigate temporal existence, grappling with the meaning of past events and experiences that are definitive of one’s life in order to come to a better understanding of the goods that are worth pursuing in the future. Taylor writes: ‘.. making sense of my present action (..) requires narrative understanding of my life, a sense of what I have become which can only be given in a story. And as I project my life forward and endorse the existing direction or give it a new one, I project a future story, not just a state of the momentary future but a bent for my whole life to come’ ([30] 48).

Narrative quests are lived, they connect to a history of (shared) experiences and they function to create coherence towards the future. The constituents of narrative quests, as well as their characteristic storylines, have therefore been imagined in different ways by different authors. Taylor just states very generally that people pursue the good life in those quests, individually as well as socially; but in MacIntyre’s work, narrative quests serve to realize excellence in a specific social or professional role, or as a human being in general. This MacIntyrian interpretation of the content of a quest as a search for excellence has, however, been criticized by feminist philosophers such as Margaret Walker, who suspects that talk about excellence equals morality to the development of ‘career selves’, which fails to appreciate capacities such as flexibility and resilience needed to deal with the ‘complexity of human lives, hopes and cares’ ([35] 120/121). Most versions of feminist ethics of care and narrative medicine take a very different attitude to narrative quests; they diversify the moral language used in a specific political context by means of the introduction of a care-vocabulary into it [2, 23], or they highlight the moral significance of specific responses to experiences of loss and suffering. Narrative medicine scholar Arthur Frank, for example, states that quest narratives primarily function to communicate an understanding of suffering as a constituent of life and to bear witness to a transformation of the character of an individual person who has to develop (partly moral) capacities to deal with it ([10] 128). He considers telling such quest narratives itself a moral act, because in dialogue relationships are intensified, but also because this type of experiences is made accessible as a cultural resource that can be used to find answers to the question how to live. While these illness stories are very personal, they always root in society and serve an individual as well as a societal purpose.

While the content of a quest has acquired different forms, there has been a lot of debate about the extent to which the historical rootedness of such quests imposes restrictions on the agency that people can develop in shaping it. As people who deviate too much from tradition cease to be sensible discussion partners (Taylor wrote, they are ‘outside our space of interlocution’), the question arises whether and how much freedom individual agents actually have to revise traditions. This is partly understood as a political question. Feminist philosopher Susan Moller Okin, for example, made a distinction between two meanings of tradition in MacIntyre’s work; ‘tradition’ as a ‘defining context, stressing the authoritative nature of its ‘texts” and tradition as ‘living’, as a ‘not yet completed narrative’, as an argument about the goods that constitute the tradition’ ([22] 61). While she could live with the last, she suspects that authors who use the word ‘tradition’ might eventually defend the first, which leaves little room for moral reformation in a narrative quest.

These two concepts of tradition have played an important role in the debate about agency in the field of narrative ethics. Liberal philosopher Jeffrey Stout uses them and writes that they go together with two different ways to deal with moral deviance in a particular society. Those who consider a tradition a ‘defining context’ tend to be more conservative and seek a managerial response to individuals with deviating opinions, such as by denying them the right to participate in decision making (or the right to vote), denying them promotions, excluding them as members of the community, or imposing censorship on their views. Those who take tradition to be ‘living’, by contrast, choose to approach tradition in a way that Stout calls ‘liberal’; they are open and tolerant to individuals with deviating views and arguments and seek dialogue with them because they understand the moral project of the tradition to be a ‘not yet completed narrative’ and therefore another person may be able to teach them something. Stout himself thinks that both approaches are possible, in practice, because society can become more traditional or liberal depending on how it decides to deal with differences of opinion. Stout himself argues for dedicated support to the project of loosening up the managerial attitude towards deviant opinions and to instead solve moral disagreement ‘democratically and dialogically’([26] 136).

Sabina Lovibond makes a similar distinction between a conservative and a liberal stance towards inherited moral distinctions in an earlier work, but adds to the political discussion also an epistemological layer ([14] 173–175). According to her, engagement in an open dialogue, and being able to judge whether rival opinions are properly argued for, presupposes the existence of institutions which in principle take a management attitude towards difference of opinion. Children, and to some extent other newcomers in society, are at first initiated into the moral distinctions that regulate interactions in a particular society. In the course of their moral upbringing, training or education, they are ‘managed’ or ‘coerced’ in the sense that any of their deviant uses of evaluative words such as ‘good’, ‘bad’, ‘just’ and ‘recommendable’ is corrected or even punished ([14] 60/61). Paradoxically, however, it is by being forced to learn these moral distinctions as well as the basic skills of sound argumentation, that people also acquire the capacity to pursue their own moral quest in which they might preserve past moral wisdoms, but also correct them based on newly acquired beliefs and experiences. Becoming able to pursue a narrative quest is therefore made possible by institutions that at the same time constrain them. This means that each adult person who engages in a quest may encounter obstacles in their environment (as in conservative institutions) but also within themselves towards change, for too much deviation from the past may undermine their own feeling of personal stability. People can be trained to become less fearful of that instability, and become open towards the opinions of others and to cultivate tolerance towards difference of opinion. This is what is typically done in liberal societies. But in a conservative tradition, individual as well as social stability is expected to depend on the authority of traditional moral distinctions and therefore deviant opinions will receive a management response.

In *Ethical formation* [15], Lovibond adds an interesting elaboration of the type of criticism of the past that this liberal view would invite. As every individual is shaped by a particular moral upbringing (hence, the title *Ethical formation*), which enables him or her to think about morality but also constrains thoughts, Lovibond thinks that a liberal criticism should be a ‘determinate critique’ ([15] 144). A determinate critic stays tied to his or her historical past to the extent to which it provides a narrative quest with a starting point and the narrator with basic argumentative and dialogical skills. A determinate criticism can be recalcitrant towards traditional values and norms and challenge them incessantly, but it will never become like anarchism. An anarchist critic abandons the established moral order entirely because it considers it to be expressive of a clash of forces rather than of rational argumentation ([15] 145). Whether to take the attitude of a determinate critic, or of an anarchist, however, is the object of a choice, for a tradition always at the same time uses power as well as arguments and the two are interdependent. A determinate critic chooses to concentrate on whether or not good arguments can be given for present moral constellations, looking at past morality as a collection of answers to specific queries and looking for ways to improve those answers towards a future of yet unrealized experience. As such, it is distinctive for determinate critics to attempt to distinguish between legitimate and illegitimate forms of authority in society, based on whether or not reasonable arguments can be provided for it. While it is always possible to take a conservative or anarchist perspective towards tradition and highlight the power-play in it, determinate critics choose to contribute to making a tradition ‘living’ and supportive of a liberal society, which allows and encourages open and argumentative discussions about whether or not moral distinctions of the past are worth preserving or not.

## Suggestions for a lay ethics quest

This debate in narrative ethics, written down much too briefly here, gives some threads to think about whether and how the past can be relevant for decision-making which shapes the future in lay ethics, and how cannot be. It gives reason to say that the ‘archetypical’ stories, which transcend the utterances of participants in focus groups considering the nanotechnological future in the present, do not in themselves provide reasons to make choices which give shape to the future. While the archetypical stories certainly offer a rich and varied perspective to the values that play a role in the public’s considerations, they do not reveal whether it would be good or bad to act on them (or some other type of story told in society) towards the future.

Davies, Macnaghten and Kearnes tend to see it as an authority problem if the stories that lay ethicists tell do not influence decision making, and plead for an institutional change which makes policy makers more responsive to the stories of lay ethicists ([19] 14). This is a valuable point, for a critical debate on the dominant evaluative distinctions of the past can only thrive if dialogue and exchange of opinions is cultivated in society. The institutional change that they defend fits into the call for a liberal approach to debate, which Stout and Lovibond also defended, and which demands a tolerant and open attitude to difference of opinion.

But choosing a narrative approach, it seems, would also invite to think more about how lay ethics interventions ought to be shaped to make room for liberal story-telling. A story may be more or less successful in influencing policy, which may depend on many factors, such as the attentiveness (or resistance) of the recipients of a story, the storytelling skills of the teller (who may be a rhetoric talent or a hesitant suggestor), the level of detachment or embeddedness of the story in concrete experiences of tellers (which may or may not be shared by the listeners) and the ordering (or disordering) of the storyline in time, as well as the (moral) acceptability of the message for a specific public. But quite apart from the question whether or not a story is successfully communicated and listened to, which a narrative perspective may help to analyze and understand better, it is important to think about what kind of tellership needs to be promoted in participants in lay ethics interventions.

As it is the purpose of lay ethics to not only collect a broad perspective to value, but to also to empower members of the public to shape the technological future, it may be advisable to invite them to take part in a narrative quest. This requires to position them as active storytellers; agents who are agents in a quest. The difficulty with the ‘archetypical stories’ is that they are re-told, and that by retelling them lay ethicists do not act as storytellers who pursue a quest, for they do not do suggestions as to how discrepancies between the diverse (counter-) narratives inherited from the past could be reconciled to shape a course of action towards the future. Pursuing a quest, means accepting the ambiguous and conflicting stories (which may include archetypical ones) which generated historically as part of one’s past and taking a role in trying to make them more coherent in an intelligible storyline that sketches a desirable development towards the future. Asking lay ethicists to act like pursuers of a quest who help to shape the future would demand to invite them to accept agency and consider conflicting stories about science that are part of their history and which inform present experiences and ask them which elements are worth preserving, and why, in the face of the choices that have to be made towards the future.

On the basis of a narrative ethics background, several suggestions may be done to encourage lay ethicists’ engagement in such a quest. As part of a narrative quest, lay ethicists should be asked, first, to reflect on a specific question or choice that relates to their experience as a person and a citizen. This is what Ferrari and Nordmann have also suggested in their critical response to the lay ethics interventions by Davies and Macnaghten [9]. Such questions could be very general and invite participants to reflect on whether and how nanotechnology research can contribute to the good life or the good society, such as: do you think nanotechnology should help to alleviate needs, and which/whose needs deserve priority? Or they could be asked to reflect on specific visions of the relation between science and society and be asked to reflect on the question: what should the role of science and technology development in society be, and how can present nanotechnology research contribute to that?

But participants can also be invited to reflect on more concrete issues such as: what application would you as individual prefer (and why)? What application deserves priority in society (and why)? Or more reflectively: do you think possibilities for human enhancement, enabled by nanotechnology, allow you to become a better or a worse person? (How) would it change your relation to other people, and would you value that? And (how) would it change your relation to society as a whole, and would you like that? Again, questions can be made even more specific when they are narrowed down further to make them contribute to concrete research; they can be asked to provide input to the list of requirements for a technological product, which researchers often develop prior to testing it; or members of the public could inform the selection of outcome measures for tests on people, as in patient trials.

In all of these ways, the question makes nanotechnology relevant for the lives of lay ethicists, which will make it more likely that they integrate it into a quest. If they have to give an answer to such questions, participants cannot simply repeat past ‘archetypical’ stories; they at the very least have to make the stories relevant for the question at hand, which means that they have to pick and choose what elements of the story they would want to use to provide an answer. This brings us to the second ingredient of narrative quests: argumentation. As part of a narrative quest, lay ethicists would have to be invited to *provide arguments* for their position; even if they draw on past stories, they would have to argue why they think these stories or these elements of stories are deserving and provide a good perspective on the issue at hand. Participants in lay ethics interventions need to take a personal perspective to the stories they inherited and argue towards other participants why they deserve preservation or alternatively, change.

Argumentation is needed, also, because archetypical stories are not the only starting point for fruitful ethical reflection. Society offers plenty of resources to start reflection. Therefore, anyone who chooses to repeat either an Enlightenment story of scientific progress or an age-old story about Pandora’s Box has something to explain. While not all narrative ethicists who use the concept of a narrative quest take tradition to be equally heterogeneous, it seems to be fair to defend such a perspective here, given the richness and variety of views about technological innovations in contemporary democracies.^4^ The third element of a narrative quest would therefore require to do effort to provide diverse input to the reflection of lay ethicists, which does justice to a variety of stories and meanings available in the social world and which allows participants to pick and choose the elements that they want to build on for the future.

Some authors proposed to do effort to diversify the input for reflection about the technological future. As the future is the object of imagination, some they proposed to do more effort to enhance lay ethicists’ imagination [29]. Marianne Boenink, Tsjalling Swierstra and Dirk Stemerding have, for example, sketched alternative, fictional, moral worlds in their jointly written articles [1, 27–28]. These moral fictions take the form of techno-ethical scenarios and allow to imagine how a society, and its morality, might develop as an effect of the introduction of a new technology into it. These techno-ethical scenarios are proposed as ‘tools’ to loosen people’s connection to their cultural heritage and help them to imagine different socio-moral possibilities ([1] 2). They encourage to leave usual moral stances behind, at least temporarily, in order to imagine alternative moral possibilities*,* or, in Swierstra’s words, they encourage to ‘…jump our ‘moral shadows’ as far as we can’ ([28] 3).^5^

Another second way to diversify the input that lay ethics take into account, can be found in card-based methodologies to enhance ethical reflection of members of the public [7]. Narratives, in this approach, are understood to generate from a conversation; therefore, they never possess a nicely polished, or entertaining, storyline, such as the archetypical stories or the techno-ethical scenarios; narrative is developed in real time in a conversation between discussion partners and therefore it often has a searching, unstable, uncertain, multiple and even ‘unruly’ character [6]. The card-based approach to such lay ethics interventions is developed by Ulrike Felt, Simone Schumann, Claudia Schwarz and Michael Strassnig and aims to enhance imagination of participants in conversations about new technologies [7]. The cards offer diverse input to this conversation. Story cards, for example, provide perspectives of specific people about a nanotechnological application that is being discussed, such as researchers, ethicists, policy-makers, members of the industry, NGO representatives or citizens who write letters to the newspaper. Application cards introduce different possible nanoapplications, which may already be available products or which can represent possible future products. Issue cards invite participants to consider nanotechnology in a wider socio-political context and bring to attention ethical, environmental, health, economic, legal and political questions and arguments. And future cards invite participants to engage with topics concerning the co-evolution of technology and society and represent concrete visions of the nanotechnological future (showing how nano can lead to a safer, more environmentally friendly, healthier society, for example).

The content of the four types of cards is based on a variety of contemporary resources such as media reports, policy documents, websites, as well as information from qualitative interviews with relevant stakeholders such as scientists, NGO representatives and policy makers. In this way, the card game pays tribute to the heterogeneity of current society as well as the diversity of issues and choices that are in need of attention. The cards function as an ‘open resource box’; it offers input to their reflection, but also allows participants flexibility in deciding for themselves what aspects they will take into account and which ones they will ignore or (temporarily) set aside ([7] 6). Furthermore, as the game asks participants first to choose cards on their own, and then share and discuss their choices with other participants, it invites them to argue for their choices ([7] 17).

In closing off this article, it is suggested that maybe the evaluative contribution of lay ethicists to decision-making at future can be much greater, if these three constituents of narrative quests are taken into account. If lay ethicists are asked to (1) reflect on a specific question or choice, (2) use diverse (imaginative) input which is informative about the heterogeneity of viewpoints in society and (3) argue for their standpoints, they could be encouraged to behave more like storytellers who have a role in the narrative quest of society, and whose contribution to the ways in which the future should be shaped could become less non-committal and more insightful. While it is certainly useful to know that the reflection of lay ethicists roots in the past, this knowledge should not stifle the reflection about what lay ethics might contribute towards reflections about the future. Rather than looking at the traditional resources that lay ethicists use as expressions of their wish to continue or repeat the past, we should develop a response which is more informed about how past stories, or tradition, shapes present day reflections, and how that past can be used in better and worse ways. Narrative ethics may be a valuable resource for this. Authors such as Stout and Lovibond show that use of tradition does not have to lead to an endless repetition of past views; it may also contribute to an open substantive debate about the value of future technologies which provides input that can be used to navigate the future. It probably does not, however, become a substantive ethical debate by itself; we have to continue to do effort to make the debate open and argumentative and think about the obstacles encountered on the way which may turn such reflections into conservative repetitions of the past.

## Footnotes

1 Alfred Nordmann is one of the most prominent critics of the repetition in the public engagement exercises. Interestingly, he also gave it an ethical dimension, saying that we would be paternalists if we would judge the future based on our own current morality, which roots in history, as we know that current moral standards will probably change as an effect of the co-evolution of technology and society. Future people—including our own future selves—may have developed a very different moral outlook and it would be wrong to judge them based on what we think is morally defensible now [20].

2 It has been noted that there is a tendency in future studies to stop looking at future anticipations as offering knowledge, but to instead perceive them as offering *narratives* about the future [11, 20, 32, 24]. In the diverse fields of future studies, such as foresight, technology assessment or studies into the ethical, legal and social aspects (ELSA) of technology, narratives about the future may be studied as accounts of present people’s *understandings* of the future, which are rooted in historically generated practices, institutions and regulations [11]; or they are studied as a kind of *speech acts*, that is, as stories about a technological future that are told to an audience in order to solicit its (political, financial, scholarly) support in bringing it about [3–4, 33–34]. Or they may do both at the same time, such as Ariana Ferrari indicates when she writes that ‘the way in which we *describe* the future will determine *how* the future will be…’ ([8] 37) The relevance of stories for ethical reflection about choices, has—to my knowledge—not been studied a lot in the field of future anticipation.

3 Alasdair MacIntyre speaks about the ‘Enlightenment project’, but of course it was not a ‘project’ with a pre-formulated goal at all. It is more like a complex historical development in which some philosophers, such as Immanuel Kant and Jeremy Bentham, developed radical new ways to rethink what ethics is and what it would require people to do, which allowed individuals to find in themselves a basis to think about what good and bad is regardless of authoritative institutions in their times.

4 Alasdair MacIntyre and Charles Taylor differ for example in this respect.

5 In order to make these scenarios useful for lay ethics interventions, Swierstra, Boenink and Stemerding argue that they should not be wild phantasies ([26] 120). While they are fictions, they are the product of ‘controlled speculation’, which sketches a line of development that is ‘convincing’, ‘plausible’ or ‘likely’ to actually materialize because it is anchored in historical developments ([1] 6). A controlled speculation should be shaped in a step-by-step manner, which starts with an investigation of the present moral landscape that may be effected by the arrival of a new technology, and which is rooted in history (step 1), then it goes on to sketch moral controversies that the new technology could raise, whose characteristic tropes and patterns can be described in the scenario (step 2), and in the last phase, the future closure of the moral controversies is imagined, based on information about what aspects of morality have proved in the past to be more liable to change (step 3). Using this step-by-step approach, the authors attempt to suggest a direction of development ‘that might be considered *plausible* in the light of past and present, moral and technological developments’ ([26] 2). Only scenarios that people could imagine becoming real are considered worthy of debate.

## References

[1] Boenink M, Swierstra T, Stemerding D (2010). Anticipating the interaction between technology and morality: a scenario study of experimenting with humans in bionanotechnology. Stud Ethics, Law Technol.

[2] Bowden P (2008). Caring: Gender-sensitive ethics.

[3] Brown N, Michael M (2003). A sociology of expectations: retrospecting prospects and prospecting retrospects. Tech Anal Strat Manag.

[4] Brown N, Rappert B, Webster A (2000). Contested futures: a sociology of prospective techno-science.

[5] Davies SR, Macnaghten P (2010). Narratives of mastery and resistance: lay ethics of nanotechnology. NanoEthics.

[6] Felt U, Fochler M, Müller A, Strassnig M (2008). Unruly ethics: on the difficulties of a bottom-up approach to ethics in the field of genomics. Public Underst Sci.

[7] Felt U, Schumann S, Schwarz CG, Strassnig M (2014). Technology of imagination: a card-based public engagement method for debating emerging technologies. Qual Res.

[8] Ferrari A (2010). Developments in the debate on nanoethics: traditional approaches and the need for new kinds of analysis. NanoEthics.

[9] Ferrari A, Nordmann A (2010). Beyond conversation: some lessons for nanoethics. NanoEthics.

[10] Frank AW (2013). The wounded storyteller: body, illness, and ethics.

[11] Grunwald A (2014) The hermeneutic side of responsible research and innovation. J Responsible Innov 1(3):274–291

[12] Kearnes M, Macnaghten P, Davies SR (2014). Narrative, nanotechnology and the accomplishment of public responses: a response to Thorstensen. NanoEthics.

[13] Kiran AH, Oudshoorn N, Verbeek PP (2015). Beyond checklists: toward an ethical-constructive technology assessment. J Responsible Innov.

[14] Lovibond S (1983). Realism and imagination in ethics.

[15] Lovibond S (2009). Ethical formation.

[16] MacIntyre A (1984). After virtue.

[17] MacIntyre AC (1988). Whose justice? Which rationality?.

[18] Macnaghten P (2010). Researching technoscientific concerns in the making: narrative structures, public responses and emerging nanotechnologies. Environ Plan.

[19] Macnaghten P, Davies SR, Kearnes M (2015) Understanding public responses to emerging technologies: a narrative approach. J Environ Policy Plan. doi:10.1080/1523908X.2015.1053110

[20] Nordmann A (2014). Responsible innovation, the art and craft of anticipation. J Responsible Innov.

[21] Oerlemans AJM, van Hoek MEC, van Leeuwen E, van der Burg S, Dekkers WJM (2013). Towards a richer debate on tissue engineering: a consideration on the basis of NEST-ethics. Sci Eng Ethics.

[22] Okin SM (2008). Justice, gender, and the family.

[23] Ruddick S (1989). Maternal thinking; towards a politics of peace.

[24] Selin C (2008). The sociology of the future: tracing stories of technology and time. Sociol Compass.

[25] Scully JL, Shakespeare T, Banks S (2006). Gift not commodity? Lay people deliberating social sex selection. Sociol Health & Illness.

[26] Stout J (2009). Democracy and tradition.

[27] Stemerding D, Swierstra T, Boenink M (2010). Exploring the interaction between technology and morality in the field of genetic susceptibility testing: a scenario study. Futures.

[28] Swierstra T, Stemerding D, Boenink M, Sollie P, Düwell M (2009). Exploring techno-moral change: the case of the obesity pill. Evaluating new technologies.

[29] Swierstra T, Rip A (2007). Nano-ethics as NEST-ethics: patterns of moral argumentation about new and emerging science and technology. NanoEthics.

[30] Taylor C (1989). Sources of the self: the making of the modern identity.

[31] Thorstensen E (2014). Public involvement and narrative fallacies of nanotechnologies. NanoEthics.

[32] Van der Burg S (2014). On the hermeneutic need for future anticipation. J Responsible Innov.

[33] Van Lente, H. V. (1993) Promising technology: the dynamics of expectations in technological developments (Doctoral dissertation, University of Twente)

[34] Van Lente HV, Rip A, Disco C, Van der Meulen BJR (1998). Expectations in technological developments: an example of prospective structures to be filled in by agency. Getting new technologies together.

[35] Walker MU (1998). Moral understandings: a feminist study in ethics.

